# A Study on Genetic Variants of Fibroblast Growth Factor Receptor 2 (*FGFR2*) and the Risk of Breast Cancer from North India

**DOI:** 10.1371/journal.pone.0110426

**Published:** 2014-10-21

**Authors:** Sarah Siddiqui, Shilpi Chattopadhyay, Md. Salman Akhtar, Mohammad Zeeshan Najm, S. V. S. Deo, N. K. Shukla, Syed Akhtar Husain

**Affiliations:** 1 Department of Biotechnology, Jamia Millia Islamia, New Delhi, India; 2 Department of Surgical Oncology, All India Institute of Medical Sciences, New Delhi, India; West Virginia University, United States of America

## Abstract

Genome-Wide Association Studies (GWAS) have identified Fibroblast growth factor receptor 2 (*FGFR2*) as a candidate gene for breast cancer with single nucleotide polymorphisms (SNPs) located in intron 2 region as the susceptibility loci strongly associated with the risk. However, replicate studies have often failed to extrapolate the association to diverse ethnic regions. This hints towards the existing heterogeneity among different populations, arising due to differential linkage disequilibrium (LD) structures and frequencies of SNPs within the associated regions of the genome. It is therefore important to revisit the previously linked candidates in varied population groups to unravel the extent of heterogeneity. In an attempt to investigate the role of *FGFR2* polymorphisms in susceptibility to the risk of breast cancer among North Indian women, we genotyped rs2981582, rs1219648, rs2981578 and rs7895676 polymorphisms in 368 breast cancer patients and 484 healthy controls by Polymerase chain reaction-Restriction fragment length polymorphism (PCR-RFLP) assay. We observed a statistically significant association with breast cancer risk for all the four genetic variants (*P*<0.05). In per-allele model for rs2981582, rs1219648, rs7895676 and in dominant model for rs2981578, association remained significant after bonferroni correction (*P*<0.0125). On performing stratified analysis, significant correlations with various clinicopathological as well as environmental and lifestyle characteristics were observed. It was evident that rs1219648 and rs2981578 interacted with exogenous hormone use and advanced clinical stage III (after Bonferroni correction, *P*<0.000694), respectively. Furthermore, combined analysis on these four loci revealed that compared to women with 0–1 risk loci, those with 2–4 risk loci had increased risk (OR = 1.645, 95%CI = 1.152–2.347, *P* = 0.006). In haplotype analysis, for rs2981578, rs2981582 and rs1219648, risk haplotype (GTG) was associated with a significantly increased risk compared to the common (ACA) haplotype (OR = 1.365, 95% CI = 1.086–1.717, *P* = 0.008). Our results suggest that intron 2 SNPs of *FGFR2* may contribute to genetic susceptibility of breast cancer in North India population.

## Introduction

Worldwide, breast cancer is the most commonly diagnosed cancer and the leading cause of cancer mortality among women [1]. Asian countries have witnessed greatest increase of the globally rising breast cancer burden during the last several decades [2]–[6]. A similar trend has been observed in India [7]–[10] with a reported 0.5–2% per annum rise in incidence across all regions and in all age groups, particularly in younger age groups (<45 years) [11]. Further, it is predicted that breast cancer cases would increase by 26%, majorly in developing countries, by 2020 [12].

Breast carcinogenesis involves a complex combination of genetic, environmental as well as lifestyle factors. Inherited susceptibility makes an important contribution to breast cancer development and the risk is around two times more in first degree relatives of women with the disease [13]. Rare mutations in several high-penetrance genes like BReast CAncer genes (*BRCA1*, *BRCA2*) account for less than 25% of the familial breast cancer risk, and less than 5% of the overall risk [14], [15]. Therefore, common variants present in other low penetrance genes may be more imperative and contribute to breast cancer along with lifestyle and environmental factors [16]. However, all of the common low risk variants described so far collectively account for <10% of the familial risk of breast cancer [17]–[25], leaving ample room for uncovering additional variants that confer risk of this disease and account for the genetic basis of the remaining major breast cancer fraction. Single nucleotide polymorphisms are the most common type of germline variations present in at least 1% of a population [26]. The effect of an individual SNP is usually small, but combinations of relevant SNPs across the genome may additively contribute to higher risk in a polygenic model [27]. Though supposed to be functionally insignificant, current evidence emphasizes their predominantly unexplored functional relevance [28]–[30].

Fibroblast Growth Factor Receptor 2 (FGFR2) belongs to the FGFR family of tyrosine kinase receptors and contributes to the process of tumorigenesis through cell growth, invasiveness, motility and angiogenesis [31]. It plays an important role during mammary gland development [32] and aberrant FGF signaling has been associated with the pathogenesis of multiple types of cancer [33]–[36]. FGFR2 overexpression has been observed in breast cancer cell lines and breast tumor tissues [37]–[38]. Human *FGFR2* gene, is located at chromosome 10q26, and contains 22 exons [39]. Two large Genome-Wide Association Studies (GWAS) have identified intron 2 SNPs of *FGFR2* to be associated with breast cancer risk, rs2981582 and rs1219648 were the most strongly associated marker SNPs in the two studies respectively [17], [18]. Association of these variants with breast cancer has been evaluated in different ethnic regions with inconsistent findings [40]–[54]. Recent meta-analysis suggests their association with breast cancer risk in Caucasian and East Asian populations [55]. Both rs2981582 and rs1219648 fell in a 25 kb linkage disequilibrium (LD) block within intron 2 region of *FGFR2* [17], [18]. Multiple haplotypes carrying the minor allele of rs2981582 were found to be associated with the risk in haplotype analysis [17]. Six polymorphisms including rs7895676 and rs2981578 were identified as potentially causal for breast cancer, with rs7895676 exhibiting strongest association in the combined analysis of European and Asian datasets [17]. Further analysis by Meyer et al. [56] support their functional relevance in relation to breast cancer risk. However, the association of rs2981578 and rs7895676 with breast cancer susceptibility still remains inconclusive [49]–[52], [57].

Wide variations in genetic architecture, including differential allele frequencies of SNPs and differently evolved LD structure for the GWAS-identified genetic variants reflect differences among ethnicities and may contribute to disparities in the incidence and characteristics of breast cancer. Thus, variants identified in one study may not have the same impact on risk in other populations. Therefore, there is a need to replicate previously associated loci in multiple populations worldwide. This will help in determining the genetic heterogeneity among different population groups for these loci, particularly in India, which witnesses a rapidly rising breast cancer burden but relatively fewer studies to identify the common breast cancer associated variations. Such studies will assist in evaluating the generalizability of initial findings and to identify the causal variants. Therefore, we tried to assess the impact of *FGFR2* intron 2 polymorphisms (rs2981582, rs1219648, rs2981578 and rs7895676) on sporadic breast cancer and determined their association with the risk for North Indian women in a case control approach, including combined effect of these variants, LD structure measurement, haplotype analysis, as well as relation with patients' clinical, environmental and lifestyle characteristics. We observed significant association of these variants with breast cancer susceptibility for North Indian women.

## Materials and Methods

### Ethics Statement

The study was approved by Institution Ethics Committee of All India Institute of Medical Sciences (AIIMS), New Delhi and the Institutional Human Ethical Committee of Jamia Millia Islamia, New Delhi. All the participants provided their written informed consent to be included in the study.

### Study subjects and specimen collection

This hospital-based case control study included a total of 852 genetically unrelated women subjects of North Indian ethnicity comprising 368 sporadic breast cancer cases and 484 healthy controls. Controls were frequency- matched to cases on age (±2 years) and geographical location. The study participation response rates for cases and controls were 88.46% and 81.07%, respectively. All breast cancer cases (aged 24–80 years) were newly diagnosed, histopathologically confirmed with primary breast cancer and were recruited from the Department of Surgical Oncology, AIIMS. Classification of breast cancer has been done according to TNM staging system by American Joint Committee on Cancer (AJCC) and Nottingham grading system for histological grading. Exclusion criteria included in the study were reported previous cancer history, metastasized cancer from other organs and previous exposure to radiotherapy or chemotherapy.

Detailed information on clinical profiles for cases and controls were collected from their medical records and are presented in Table S1. Included were tumor characteristics [age at diagnosis, tumor size, lymph node (LN) involvement, clinical stage, histological grade, estrogen receptor (ER) status, progesterone receptor (PR) status and human epidermal growth factor receptor 2 (HER2) status], reproductive history [including age at menarche, menopausal status, age at menopause, parity, age at first live birth and status of breastfeeding] as well as several demographic, lifestyle and environmental factors [Exogenous hormone use for purposes like contraception/infertility treatment/hormone replacement therapy (yes: for >6 months/No: for ≤6 months), BMI (Basal metabolic index, calculated as weight divided by squared height, kg/m**^2^**), geographical location, education level and economic independence (employed/unemployed)].

### Extraction of genomic DNA

Participating women provided 3–5 ml of venous blood samples used for isolating genomic DNA based on standard phenol–chloroform extraction method [58]. DNA samples were stored at −80°C until used for further analysis.

### SNP selection and Genotype analysis

Previously reported *FGFR2* SNPs showing association with breast cancer in one or more GWAS and candidate gene studies including the two proposed functional variants (rs2981582C/T, rs1219648A/G, rs2981578A/G, rs7895676T/C) [17], [18], [40]–[52], [56] were selected for genotyping. All the four SNPs were analyzed using the Polymerase chain reaction-restriction fragment length polymorphism (PCR-RFLP) assay. Details of selected SNPs, primer sequences used for PCR, sizes of the PCR products, restriction endonucleases (New England Biolabs, USA) used for digestion and their recognition sequences, as well as size of various digested fragments obtained distinguishing different genotypes for all the four SNPs are described in Table 1 and Table S2. For all the SNPs, restriction digested fragments were subjected to analysis by 2–3.5% agarose gel electrophoresis. In order to validate the data generated by PCR-RFLP assay method, 5% of randomly selected samples were directly sequenced. DNA sequencing was carried out at Xcelris Labs Ltd., India. The quality of genotyping was assessed by re-genotyping 10% of randomly selected samples; no discrepancy in the replicate genotyping could be obtained.

**Table 1:** Primers used for genotyping *FGFR2* SNPs.

FGFR2 SNPs	Primer Sequences	PCR product length (base pairs)	Restriction endonucleases used	Genotype (size of digested fragments in base pairs)
rs2981582	F 5′CGTGAGCCAAGCCTCTACTT3′			CC (140, 84, 38)
	R 5′TAAGTGTGCTGTTCATTCA3′	262	AciI	CT (178, 140, 84, 38)
				TT (178, 84)
rs1219648	F 5′ATGGTACCGGTTTCCCAA3′			AA (180)
	R 5′TGTGATTTGTATGTGGTAG3′	180	BspQI	AG (180, 106, 74)
				GG (106, 74)
rs2981578	F 5′CCCAGAAAGCCTACATTCGT3′			AA (330)
	R 5′CAGGACCCAAGGAAGGCAG3′	330	AciI	AG (330, 182, 148)
				GG (182, 148)
rs7895676	F 5′AGGTGCGGTGGCTCATGTCTGTA3′			TT (292, 54)
	R 5′CTGACTTCAATGGCGGGACTCCAT3′	346	DpnII	CT (292, 175, 117, 54)
				CC (175, 117, 54)

### Statistical analysis

To compare the overall distribution of genotypes between patients and healthy controls 3×2 Chi-square (χ2) test was performed. Hardy–Weinberg equilibrium (HWE) was evaluated by a goodness-of-fit χ2 test. For estimating associations between individual genotypes and breast cancer risk, and for cumulative risk analysis, odds ratios (ORs) and their 95% confidence intervals (CIs) were computed using unconditional logistic regression analysis with adjustment for age. One-way ANOVA (Analysis of variance) was carried out for estimating the contribution of different number of risk loci to breast cancer risk. These statistical analysis were performed using Statistical Package for the Social Sciences, version 17 (SPSS Inc., Chicago, IL, USA) and *P*<0.05 was considered statistically significant. Further, all *P*-values were corrected for multiple comparisons according to Bonferroni method. LD pattern and population haplotype frequencies for the SNPs were estimated using HaploView v4.2 [59]. Fisher's exact test was performed for determining the association of haplotypes with diseased condition.

## Results

Results of genotype analysis on the four selected *FGFR2* intronic variants (rs2981582C/T, rs1219648A/G, rs2981578A/G, and rs7895676T/C) were available from 368 breast cancer cases/484 healthy controls and a notably significant association with breast cancer susceptibility was observed.

### Hardy–Weinberg equilibrium testing

The observed genotype frequencies were found to be in agreement (χ2 test, *P*>0.05) with Hardy–Weinberg equilibrium in both cases (*P* = 0.545, 0.261, 0.347 and 0.832) and controls (*P* = 0.526, 0.569, 0.278 and 0.467) for the SNPs rs2981582, rs1219648, rs2981578 and rs7895676 respectively.

### FGFR2 SNPs and overall breast cancer risk

Distribution of genotype and allele frequencies of the four *FGFR2* SNPs in breast cancer cases and controls are shown in Table 2. Chi-square test depicted a significant association for the four *FGFR2* variants with overall breast cancer risk (*P*<0.05). Logistic regression analysis (age adjusted) further confirmed this association which remained significant in per-allele model for rs2981582/T, rs1219648/G, rs7895676/C and in dominant model for rs2981578 (AG+GG) even after Bonferroni correction (*P*<0.0125).

**Table 2:** Genotype and allele frequencies of *FGFR2* polymorphisms in sporadic breast cancer cases and controls.

Genotypes	Cases (N = 368)	Controls (N = 484)	a *P*	b *P*	aOR (95% CI)
*FGFR2* (rs2981582)					
CC	144 (39.13%)	226 (46.69%)		-	1.000 (referent)
CT	168 (45.65%)	205 (42.36%)		0.084	1.300 (0.965–1.752)
TT	56 (15.22%)	53 (10.95%)	0.045	0.022	1.668 (1.078–2.582)
CC vs. CT+TT				0.025	1.379 (1.042–1.824)
C (%)	61.96	67.87		-	1.000 (referent)
T (%)	38.04	32.13		0.011*	1.297 (1.061–1.586)
*FGFR2* (rs1219648)					
AA	110 (29.89%)	183 (37.81%)		-	1.000 (referent)
AG	192 (52.17%)	234 (48.35%)		0.042	1.375 (1.012–1.867)
GG	66 (17.93%)	67 (13.84%)	0.036	0.019	1.644 (1.084–2.495)
AA vs. AG+GG				0.015	1.435 (1.073–1.920)
A (%)	55.98	61.98		-	1.000 (referent)
G (%)	44.02	38.02		0.012*	1.282 (1.055–1.558)
*FGFR2* (rs2981578)					
AA	54 (14.67%)	105 (21.69%)		-	1.000 (referent)
AG	185 (50.27%)	228 (47.11%)		0.019	1.581 (1.079–2.315)
GG	129 (34.05%)	151 (31.20%)	0.033	0.014	1.661 (1.108–2.489)
AA vs. AG+GG				0.009*	1.613 (1.124–2.314)
A (%)	39.81	45.25		-	1.000 (referent)
G (%)	60.19	54.75		0.025	1.249 (1.029–1.518)
*FGFR2* (rs7895676)					
TT	71 (19.29%)	124 (25.62%)		-	1.000 (referent)
TC	179 (48.64%)	234 (48.35%)		0.097	1.349 (0.947–1.923)
CC	118 (32.07%)	126 (26.03%)	0.043	0.011*	1.649 (1.119–2.431)
TT vs. TC+CC				0.027	1.455 (1.044–2.029)
T (%)	43.61	49.79		-	1.000 (referent)
C (%)	56.39	50.21		0.011*	1.282 (1.058–1.555)

*P<0.0125, *P* values significant after Bonferroni correction.

*OR* odds ratio, *CI* confidence interval.

a *P* value for 3×2 χ2 test of comparison of overall genotype frequencies between cases and controls.

b *P* value and corresponding age-adjusted OR (aOR) with 95% CIs [aOR (95% CI)] for comparison of genotype frequencies between cases and controls by logistic regression analysis (age is not adjusted in allele frequency comparisons).

To evaluate the cumulative risk for these SNPs, we categorized study subjects as carrying 0 risk loci, 1 risk loci, 2 risk loci, 3 risk loci and 4 risk loci (risk loci represents presence of risk allele at SNP position). For determining the contribution of different risk loci overall as well as in four different trio combinations of these SNPs, logistic regression and one-way ANOVA tests were performed (Table 3). Logistic regression analysis revealed a significantly higher risk in carriers with 2–4 risk loci (aOR = 1.645, 95%CI  = 1.152–2.347, *P* = 0.006) compared to those with 0–1 risk loci. Further a progressively increased risk was noted from 1 risk loci (aOR  = 1.600, 95%CI = 0.754–3.394) to 2 risk loci (aOR  = 1.786, 95%CI  = 1.076–2.964) to 3–4 risk loci (aOR  = 1.855, 95%CI  = 1.230–2.799) in comparison to 0 risk loci (Table S3). ANOVA determined significant (*P*<0.05) differences in the contribution of various risk loci to diseased condition for the four SNPs taken together as well as in different combinations (***^c^*** *P* values in Table 3). Moreover, logistic regression analysis revealed significant contribution of only two SNP combinations for breast cancer risk considering dichotomized 2–3 risk loci compared to 0–1 risk loci, including ABD (rs7895676, rs2981578 and rs1219648; aOR = 1.622, 95%CI  = 1.150–2.289, *P* = 0.006), and BCD (rs2981578, rs2981582 and rs1219648; aOR = 1.431, 95%CI = 1.065–1.923, *P* = 0.018). On conducting multiple comparison analysis in ANOVA, similar trends were observed (Table S3, Table S4). Significant association with the risk was noted for 2 risk loci (*P* = 0.026) and 4 risk loci (*P* = 0.002) compared to 0 risk loci. Also for the four different SNP combinations, significant *P* values for all the risk loci were observed for combination BCD (***^b^*** *P* values in Table 3) indicating its predominant contribution towards risk.

**Table 3:** Estimated risk of combined *FGFR2* SNPs (rs7895676, rs2981578, rs2981582 and rs1219648).

SNP Combi-nations	No. of risk loci	No. of risk loci (dichotomized)	No. of cases (%)	No. of controls (%)	aOR (95% CI)	a *P* value	b *P* value	c *P* value
dABCD	0						-	
	1	0–1	56 (15.22)	110 (22.73)	1.000 (Referent)		0.234	
	2						0.026	
	3						0.115	
	4	2–4	312 (84.78)	374 (77.27)	1.645 (1.152–2.347)	0.006	0.002	0.037
ABC	0						-	
	1	0–1	69 (18.75)	116 (23.97)	1.000 (Referent)		0.013	
	2						0.086	
	3	2–3	299 (81.25)	368 (76.03)	1.375 (0.982–1.925)	0.064	0.002	0.007
BCD	0						-	
	1	0–1	105 (28.53)	175 (36.16)	1.000 (Referent)		0.027	
	2						0.002	
	3	2–3	263 (71.47)	309 (63.84)	1.431 (1.065–1.923)	0.018	0.003	0.011
ABD	0						-	
	1	0–1	62 (16.85)	119 (24.59)	1.000 (Referent)		0.250	
	2						0.006	
	3	2–3	306 (83.15)	365 (75.41)	1.622 (1.150–2.289)	0.006	0.006	0.028
ACD	0						-	
	1	0–1	112 (30.43)	177 (36.57)	1.000 (Referent)		0.148	
	2						0.572	
	3	2–3	256 (69.57)	307 (63.43)	1.329 (0.992–1.780)	0.056	0.006	0.029

*OR* odds ratio, *CI* confidence interval.

a *P* value and corresponding age-adjusted OR (aOR) with 95% CIs for combined risk analysis by logistic regression test.

b *P* value for association of different number of risk loci with breast cancer risk in comparison to 0 risk loci by one-way ANOVA analysis displaying multiple comparisons output.

c *P* value <0.05 represents significant difference between contribution of different number of risk loci to breast cancer risk.

d A =  rs7895676, B =  rs2981578, C =  rs2981582 and D =  rs1219648.

### FGFR2 SNPs and clinicopathological characteristics

Further we analyzed association of these variants with various clinicopathological characteristics including several reproductive and environmental risk factors of breast cancer in a stratified analytical approach (Table 4) The corrected *P* value cut-off after bonferroni correction was set as (*P*<0.000694).

**Table 4:** Association of *FGFR2* rs2981582, rs1219648, rs2981578 and rs7895676 SNPs with clinicopathological, life style and environmental characteristics of breast cancer patients from North India.

Character-istic	*FGFR2* rs2981582			*FGFR2* rs1219648			*FGFR2* rs2981578			*FGFR2* rs7895676		
	Genotype (n)	aOR (95% CI)	*P* value	Genotype (n)	aOR (95% CI)	*P* value	Genotype (n)	aOR (95% CI)	*P* value	Genotype (n)	aOR (95% CI)	*P* value
Menopaus-	CC (51/93)	1.000		AA (41/69)	1.000		AA (21/33)	1.000		TT (31/40)	1.000	0.660
al Status	CT (79/89)	0.236 (0.067-0.827)	0.024	AG (88/104)	0.251(0.069–.909)	0.035	AG (74/111)	1.733 (0.418-7.191)	0.449	TC (77/102)	0.753 (0.212–2.669)	0.660
Pre/Post	TT (25/31)	0.671 (0.178–2.535)	0.557	GG (26/40)	1.128 (0.327–3.898)	0.849	GG (60/69)	0.559 (0.109–2.857)	0.485	CC (47/71)	0.800 (0.208–3.070)	0.745
	CT+TT (104/120)	0.349 (0.124–0.980)	0.046	AG+GG (114/144)	0.470 (0.168–1.316)	0.151	AG+GG (134/180)	1.137 (0.289–4.476)	0.855	TC+CC (124/173)	0.772 (0.242–2.468)	0.663
Age at	CC (39/54)	1.0000		AA (34/35)	1.000		AA (19/14)	1.000		TT (22/18)	1.000	
Menopause	CT (42/47)	0.615 (0.327–1.157)	0.132	AG (50/54)	0.852 (0.451–1.607)	0.620	AG (50/61)	1.819 (0.802–4.123)	0.152	TC (49/53)	1.121 (0.510–2.460)	0.777
(years)	TT (17/14)	0.640 (0.267–1.534)	0.317	GG (14/26)	1.009 (0.382–2.666)	0.985	GG (29/40)	1.895 (0.791–4.539)	0.151	CC (27/44)	1.896 (0.824–4.365)	0.133
≤49/≥50	CT+TT (59/61)	0.622 (0.347–1.113)	0.109	AG+GG (64/80)	0.883 (0.485–1.608)	0.685	AG+GG (79/101)	1.848 (0.846–4.035)	0.123	TC+CC (76/97)	1.397 (0.669–2.919)	0.374
ER Status	CC (56/88)	1.000		AA (46/64)	1.000		AA (24/30)	1.000		TT (37/34)	1.000	
Positive/	CT (81/87)	1.448 (0.905–2.317)	0.122	AG (86/106)	1.062 (0.650–1.733)	0.811	AG (93/92)	1.333 (0.711–2.498)	0.370	TC (78/101)	0.677 (0.382–1.200)	0.182
Negative	TT (35/21)	3.123 (1.621–6.018)	0.001	GG (40/26)	2.183 (1.155–4.127)	0.016	GG (55/74)	1.024 (0.530–1.977)	0.944	CC (57/61)	0.871 (0.475–1.596	0.655
	CT+TT (116/108)	1.761 (1.135–2.732)	0.012	AG+GG (126/132)	1.285 (0.808–2.042)	0.290	AG+GG (148/166)	1.196 (0.657–2.177)	0.558	TC+CC (135/162)	0.751 (0.439–1.284)	0.296
PR Status	CC (59/85)	1.000		AA (48/62)	1.000		AA (25/29)	1.000		TT (29/42)	1.000	
Positive/	CT (91/77)	1.642 (1.035–2.606)	0.035	AG (102/90)	1.401 (0.863–2.272)	0.172	AG (86/99)	1.065 (0.571–1.984)	0.844	TC (101/78)	1.990 (1.118–3.544)	0.019
Negative	TT (29/27)	1.652 (0.874–3.121)	0.122	GG (29/37)	0.965 (0.516–1.805)	0.910	GG (68/61)	1.424 (0.742–2.732)	0.288	CC (49/69)	1.119 (0.606–2.064)	0.719
	CT+TT (120/104)	1.645 (1.067–2.535)	0.024	AG+GG (131/127)	1.270 (0.802–2.010)	0.308	AG+GG (154/160)	1.200 (0.663–2.170)	0.547	TC+CC (150/147)	1.573 (0.915–2.703)	0.101
HER 2 Sta-	CC (65/79)	1.000		AA (59/51)	1.000		AA (22/32)	1.000		TT (31/40)	1.000	
Tus	CT (86/82)	1.333 (0.841–2.113)	0.222	AG (88/104)	0.772 (0.476–1.253)	0.295	AG (95/90)	1.659 (0.884–3.115)	0.115	TC (91/88)	1.357 (0.765–2.406)	0.297
Positive/	TT (23/33)	0.814 (0.427–1.551)	0.532	GG (27/39)	0.595 (0.317–1.119)	0.107	GG (57/72)	1.173 (0.607–2.267)	0.635	CC (52/66)	0.983 (0.534–1.808)	0.955
Negative	CT+TT (109/115)	1.176 (0.765–1.808)	0.461	AG+GG (115/143)	0.722 (0.456–1.143)	0.164	AG+GG (152/162)	1.438 (0.790–2.619)	0.235	TC+CC (143/154)	1.190 (0.694–2.038)	0.527
Tumor size	CC(102/42)	1.000		AA (74/36)	1.000		AA (37/17)	1.000		TT (55/16)	1.000	
(cm)	CT (130/38)	1.500 (0.889–2.532)	0.129	AG (144/48)	1.574 (0.926–2.676)	0.094	AG (141/44)	1.612 (0.818–3.180)	0.168	TC (134/45)	0.880 (0.451–1.717)	0.708
>2/≤2	TT (37/19)	0.772 (0.391–1.524)	0.456	GG (51/15)	1.664 (0.818–3.386)	0.160	GG (91/38)	1.169 (0.581–2.353)	0.661	CC (80/38)	0.597 (0.300–1.191)	0.143
	CT+TT (167/57	1.247 (0.773–2.011)	0.366	AG+GG (195/63)	1.597 (0.967–2.638)	0.068	AG+GG (232/82)	1.404 (0.742–2.658)	0.297	TC+CC (214/83)	0.746 (0.399–1.395)	0.359
Lymph no-	CC (85/59)	1.000		AA (56/54)	1.000		AA (28/26)	1.000		TT (34/37)	1.000	
de Status	CT (108/60)	1.359 (0.846–2.185)	0.205	AG (132/60)	2.360 (1.424–3.910)	0.001	AG (124/61)	1.904 (1.012–3.581)	0.046	TC (123/56)	2.521 (1.402–4.532)	0.002
Positive/	TT (32/24)	0.888 (0.463–1.702)	0.721	GG (37/29)	1.275 (0.677–2.400)	0.452	GG (73/56)	1.185 (0.616–2.281)	0.610	CC (68/50)	1.434 (0.778–2.642)	0.248
Negative	CT+TT (140/84)	1.218 (0.783–1.893)	0.382	AG+GG (169/89)	1.986 (1.239–3.185)	0.004	AG+GG (197/117)	1.558 (0.858–2.827)	0.145	TC+CC (191/106)	1.984 (1.154–3.413)	0.013
Clinical	CC (86/58)	1.000		AA (58/52)	1.000		AA (19/35)	1.000		TT (43/28)	1.000	
Stage	CT (88/80)	0.783 (0.491–1.248)	0.303	AG (111/81)	1.307 (0.799–2.137)	0.287	AG (120/65)	3.588 (1.851–6.955)	0.0001	TC (102/77)	0.911 (0.507–1.639)	0.756
III+IV/I+II	TT (29/27)	0.629 (0.327–1.212)	0.166	GG (34/32)	0.936 (0.496–1.767)	0.839	GG (64/65)	1.829 (0.923–3.624)	0.083	CC (58/60)	0.614 (0.329–1.145)	0.125
	CT+TT (117/107)	0.741 (0.479–1.148)	0.180	AG+GG (145/113)	1.197 (0.751–1.908)	0.450	AG+GG (184/130)	2.698 (1.442–5.051)	0.002	TC+CC (160/137)	0.776 (0.447–1.349)	0.369
Histologic-	CC (32/93)	1.000		AA (12/87)	1.000		AA (8/39)	1.000		TT (17/43)	1.000	
al grade	CT (27/112)	0.717 (0.396–1.299)	0.273	AG (38/118)	2.378 (1.152–4.907)	0.019	AG (36/126)	1.309 (0.551–3.113)	0.542	TC (32/122)	0.540 (0.264–1.107)	0.092
III/I+II	TT (4/48)	0.282 (0.093–0.857)	0.026	GG (13/48)	2.170 (0.904–5.210)	0.083	GG (19/88)	1.069 (0.421–2.715)	0.888	CC (14/88)	0.351 (0.152–0.807)	0.014
	CT+TT (31/160)	0.594 (0.337–1.046)	0.071	AG+GG (51/166)	2.318 (1.155–4.649)	0.018	AG+GG (55/214)	1.216 (0.526–2.811)	0.647	TC+CC (46/210)	0.464 (0.235–0.916)	0.027
Age at	CC (106/38)	1.000		AA (79/31)	1.000		AA (31/23)	1.000		TT (52/19)	1.000	
Menarche	CT (108/60)	0.624 (0.379–1.027)	0.064	AG (123/69)	0.683 (0.406–1.150)	0.151	AG (120/65)	1.447 (0.771–2.716)	0.250	TC (117/62)	0.669 (0.358–1.250)	0.207
(years)	TT (34/22)	0.582 (0.299–1.134)	0.112	GG (46/20)	0.911 (0.462–1.795)	0.787	GG (97/32)	2.432 (1.227–4.821)	0.011	CC (79/39)	0.738 (0.380–1.432)	0.370
>12/≤12	CT+TT (142/82)	0.613 (0.384–0.978)	0.040	AG+GG (169/89)	0.736 (0.447–1.209)	0.226	AG+GG (217/97)	1.769 (0.969–3.227)	0.063	TC+CC (196/101)	0.696 (0.385–1.259)	0.231
Age at first	CC (25/115)	1.000		AA (16/92)	1.000		AA (7/43)	1.000		TT (16/52)	1.000	
live birth	CT (25/134)	0.979 (0.521–1.840)	0.947	AG (34/151)	1.531 (0.782–2.997)	0.215	AG (39/136)	1.790 (0.728–4.399)	0.205	TC (30/141)	0.762 (0.370–1.566)	0.459
(years)	TT (13/41)	1.313 (0.599–2.879)	0.496	GG (13/47)	1.672 (0.723-3.868)	0.230	GG (17/111)	0.904 (0.342–2.388)	0.838	CC (17/97)	0.558 (0.253–1.229)	0.147
>29/≤29	CT+TT (38/175)	1.072 (0.602–1.909)	0.812	AG+GG (47/198)	1.568 (0.827–2.973)	0.169	AG+GG (56/247)	1.380 (0.577–3.302)	0.469	TC+CC (47/238)	0.671 (0.343–1.315)	0.246
BMI	CC (41/93)	1.000		AA (26/78)	1.000		AA (11/36)	1.000		TT (27/41)	1.000	
(kg/m^2^)	CT (53/107)	1.071(0.642–1.785)	0.794	AG (61/121)	1.462 (0.837–2.553)	0.182	AG (50/121)	1.209 (0.555–2.633)	0.632	TC (50/115)	0.627 (0.338–1.165)	0.140
≥25/<25	TT (14/37)	0.819 (0.388–1.729)	0.600	GG (21/38)	1.628 (0.795–3.334)	0.183	GG (47/80)	1.783 (0.808–3.932)	0.152	CC (31/81)	0.562 (0.289–1.093)	0.090
	CT+TT (67/144)	1.005 (0.620–1.627)	0.985	AG+GG (82/159)	1.502 (0.881–2.560)	0.135	AG+GG (97/201)	1.437 (0.684–3.019)	0.339	TC+CC (81/196)	0.600 (0.336–1.070)	0.083
Parity	CC (140/4)	1.000		AA (108/2)	1.000		AA (50/4)	1.000		TT (68/3)	1.000	
Parous/Nu-	CT (159/9)	0.460 (0.131–1.619)	0.227	AG (185/7)	0.455 (0.090–2.297)	0.340	AG (175/10)	1.396 (0.392–4.974)	0.607	TC (171/8)	0.694 (0.165–2.924)	0.618
Lliparous	TT (54/2)	1.155 (0.192–6.934)	0.875	GG (60/6)	0.200 (0.038–1.058)	0.058	GG (128/1)	11.886 (1.22–115.4)	0.033	CC (114/4)	1.028 (0.210–5.030)	0.972
	CT+TT (213/11)	0.586 (0.176–1.948)	0.383	AG+GG (245/13)	0.336 (0.072–1.558)	0.163	AG+GG (303/11)	2.311 (0.669–7.990)	0.186	TC+CC (285/12)	0.810 (0.207–3.168)	0.762
Breastfeed-	CC (126/18)	1.000		AA (97/13)	1.000		AA (48/6)	1.000		TT (68/3)	1.000	
Ing	CT (155/13)	1.764 (0.807–3.857)	0.155	AG (183/9)	2.623 (1.060–6.488)	0.037	AG (169/16)	1.271 (0.458–3.527)	0.645	TC (167/12)	0.489 (0.129–1.861)	0.294
Yes/No	TT (51/5)	2.040 (0.687–6.056)	0.199	GG (52/14)	0.521 (0.222–1.223)	0.134	GG (115/14)	1.088 (0.384–3.085)	0.874	CC (97/21)	0.164 (0.045–0.602)	0.006
	CT+TT (206/18)	1.838 (0.894–3.779)	0.098	AG+GG (235/23)	1.340 (0.638–2.813)	0.440	AG+GG (284/30)	1.186 (0.456–3.084)	0.727	TC+CC (264/33)	0.283 (0.081–0.987)	0.048
Education	CC (77/65)	1.000		AA (51/58)	1.000		AA (31/23)	1.000		TT (44/27)	1.000	
Level	CT (91/72)	1.070 (0.676–1.694)	0.772	AG (103/85)	1.323 (0.817–2.143)	0.255	AG (102/81)	0.964 (0.518–1.792)	0.907	TC (88/84)	0.677 (0.381–1.204)	0.184
(years)	TT (24/32)	0.580 (0.306–1.101)	0.096	GG (38/26)	1.558 (0.830–2.926)	0.168	GG (59/65)	0.676 (0.352–1.297)	0.239	CC (60/58)	0.669 (0.365–1.227)	0.194
>12/≤12	CT+TT (115/104)	0.914 (0.596–1.402)	0.680	AG+GG (141/111)	1.381 (0.874–2.182)	0.167	AG+GG (161/146)	0.835 (0.463–1.506)	0.549	TC+CC (148/142)	0.674 (0.393–1.156)	0.152
Exogenous	CC (18/119)	1.000		AA (7/101)	1.000		AA (9/41)	1.000		TT (16/53)	1.000	
Hormone	CT (22/144)	1.037 (0.524–2.052)	0.916	AG (25/161)	2.333 (0.959–5.678)	0.062	AG (29/149)	0.947 (0.407–2.201)	0.899	TC (21/149)	0.503 (0.240–1.056)	0.069
Use	TT (13/40)	2.067 (0.910–4.696)	0.083	GG (21/41)	7.618 (2.972–19.53)	0.0001	GG (15/113)	0.636 (0.254–1.591)	0.333	CC (16/101)	0.553 (0.253–1.208)	0.137
Yes/No	CT+TT (35/184)	1.280 (0.686–2.388)	0.438	AG+GG (46/202)	3.473 (1.492–8.081)	0.004	AG+GG (44/262)	0.811 (0.362–1.817)	0.610	TC+CC (37/250)	0.524 (0.267–1.028)	0.060
Place of	CC (76/68)	1.000		AA (59/51)	1.000		AA (33/21)	1.000		TT (37/34)	1.000	
residence	CT (97/71)	1.185 (0.753–1.867)	0.463	AG (114/78)	1.254 (0.776–2.026)	0.355	AG (107/78)	0.892 (0.478–1.666)	0.720	TC (107/72)	1.373 (0.781–2.413)	0.271
Urban/Ru-	TT (34/22)	1.377 (0.727–2.608)	0.326	GG (34/32)	0.891 (0.482–1.649)	0.714	GG (67/62)	0.692 (0.361–1.328)	0.268	CC (63/55)	1.068 (0.588–1.939)	0.830
Ral	CT+TT (131/93)	1.231 (0.805–1.884)	0.338	AG+GG (148/110)	1.145 (0.727–1.802)	0.560	AG+GG (174/140)	0.803 (0.443–1.456)	0.470	TC+CC (170/127)	1.238 (0.730–2.100)	0.429
Economic	CC(66/73)	1.000		AA (49/54)	1.000		AA (19/31)	1.000		TT (33/36)	1.000	
independe-	CT (105/57)	2.036 (1.273–3.256)	0.003	AG (102/80)	1.395 (0.849–2.291)	0.189	AG (98/79)	0.713 (0.361–1.410)	0.331	TC (88/80)	1.206 (0.676–2.154)	0.526
Nce	TT (17/32)	0.557 (0.279–1.112)	0.097	GG (37/28)	1.420 (0.757-2.665)	0.275	GG (71/52)	0.540 (0.265–1.097)	0.088	CC (67/46)	1.637 (0.888–3.017)	0.114
Employed/Unemploy-ed	CT+TT (122/89)	1.495 (0.968–2.309)	0.070	AG+GG (139/108)	1.402 (0.877–2.242)	0.158	AG+GG (169/131)	0.636 (0.331–1.219)	0.173	TC+CC (155/126)	1.374 (0.800–2.361)	0.250

*P*<0.000694, *P* values significant after Bonferroni correction.

*aOR* age-adjusted odds ratio, *CI* confidence interval.

*P* value and corresponding age-adjusted OR (aOR) with 95% CIs [aOR (95% CI)] by logistic regression analysis.

For *rs2981582(C/T)/FGFR2*, genotype CT vs. CC (vs.  =  in comparison to) and combined CT+TT vs. CC significantly correlated with premenopausal status (*P* = 0.024, 0.046 respectively). T allele displayed stronger association with ER-positive women (TT vs. CC, *P* = 0.001; CT+TT vs. CC, *P* = 0.012) and with PR-positive women (CT vs. CC, *P* = 0.035; CT+TT vs. CC, *P* = 0.024). Association with histologically less malignant grade I+II (TT vs. CC, *P* = 0.026), early age at menarche (CT+TT vs. CC, *P* = 0.040) and employed status (CT vs. CC, *P = *0.003) was also observed.

For *rs1219648 (A/G)/FGFR2*, AG genotype presented a significantly higher distribution in premenopausal patients exhibiting higher risk (AG vs. AA, *P* = 0.035). Furthermore, G-carriers were more significantly linked to tumors with ER-positive status (GG vs. AA, *P* = 0.016), LN positive status (AG vs. AA, *P* = 0.001; AG+GG vs. AA, *P* = 0.004), and more malignant histological grade III (AG vs. AA, *P* = 0.019; AG+GG vs. AA, *P* = 0.018). Also, interaction of the risk allele with positive breastfeeding status (AG vs. AA, *P* = 0.037) and strongly with exogenous hormone exposure (GG vs. AA, *P = *0.0001; AG+GG vs. AA, *P = *0.004) was noted.

For *rs2981578 (A/G)/FGFR2*, G allele carriers were more likely to bear tumors of greater aggressiveness with advanced clinical stage III+IV (AG vs. AA, *P* = 0.0001; AG+GG vs. AA, *P* = 0.002) and LN metastasis (AG vs. AA, *P* = 0.046). Association with late age at menarche (GG vs. AA, *P* = 0.011) and parous status (GG vs. AA, *P = *0.033) was also observed.

For *rs7895676 (T/C)/FGFR2*, TC genotype exhibited significantly greater risk in PR-positive women (TC vs. TT, *P* = 0.019). Further association of the risk allele with LN-positive status (TC vs. TT, *P* = 0.002; TC+CC vs. TT, *P* = 0.013), pathologically less malignant grade I+II tumors (CC vs. TT, *P* = 0.014; TC+CC vs. TT, *P* = 0.027) and with negative breastfeeding status (CC vs. TT, *P = *0.006; TC+CC vs. TT, *P = *0.048) was evidenced.

### Linkage Disequilibrium (LD) and Haplotype analysis

Setting measure of high LD between two genetic markers cut off to a value r^2^ ≥0.80, D′ = 1, in control group of our study population, the four studied SNPs were found to be in moderate to weak LD (pair wise r^2^ value range from 0.175–0.680, D′ value range from 0.610–0.938, Figure S1). Haplotype frequencies were estimated for the four SNPs taken together as well as for different combinations of SNPs taken three and two at a time using Haploview and the association with the risk was determined by applying Fisher's exact test (Table 5, Table S5). Increased risk for the haplotype having only risk alleles compared to the one having only common alleles was observed for all the possible combinations (*P*<0.05). However, contrary to the combined risk analysis, predominant contribution towards the risk in terms of higher odds ratio was observed for trio combinations ABC (rs7895676, rs2981578 and rs2981582; OR = 1.422, 95%CI = 1.126–1.797, *P = *0.004) and ACD (rs7895676, rs2981582 and rs1219648; OR = 1.442, 95%CI = 1.144–1.816, *P = *0.002). However, SNP combination BCD (rs2981578, rs2981582 and rs1219648), seems to be relevant as pair wise D′>0.80 for these three SNPs (Figure S1) and carriers of GTG (carrying only risk alleles) haplotype had a significantly greater risk compared to ACA (carrying only wild-type alleles) haplotype, (OR = 1.365, 95%CI = 1.086–1.717, *P = *0.008). While among duo SNP combinations, AC (rs7895676 and rs2981582) displayed highest odds ratio (OR = 1.449, 95%CI = 1.153–1.822, *P* = 0.002), Table S5.

**Table 5:** Frequencies of inferred haplotypes of *FGFR2* SNPs rs7895676, rs2981578, rs2981582 and rs1219648 in breast cancer cases and controls.

SNP combinations	aHaplotype	Cases (N = 368)	Controls (N = 484)	OR (95% CI)	b *P* value
cABCD	TACA	0.362	0.405	1.000 (referent)	
	CGTG	0.329	0.265	1.388 (1.097–1.755)	0.007
	Others	0.309	0.330	1.053 (0.836–1.326)	0.681
ABC	TAC	0.359	0.402	1.000 (referent)	
	CGT	0.340	0.267	1.422 (1.126–1.797)	0.004
	Others	0.301	0.331	1.022 (0.811–1.289)	0.859
BCD	ACA	0.370	0.427	1.000 (referent)	
	GTG	0.340	0.287	1.365 (1.086–1.717)	0.008
	Others	0.290	0.286	1.173 (0.927–1.484)	0.187
ABD	TAA	0.365	0.405	1.000 (referent)	
	CGG	0.386	0.327	1.306 (1.045–1.632)	0.020
	Others	0.249	0.268	1.030 (0.806–1.315)	0.851
ACD	TCA	0.388	0.433	1.000 (referent)	
	CTG	0.337	0.260	1.442 (1.144–1.816)	0.002
	Others	0.275	0.307	0.996 (0.789–1.259)	1.000

*OR* odds ratio, *CI* confidence interval.

a Haplotype In the order of *FGFR2* SNPs rs7895676, rs2981578, rs2981582, rs1219648.

b *P* value and corresponding OR with 95% CI for Fisher's exact test.

c A =  rs7895676, B =  rs2981578, C =  rs2981582 and D =  rs1219648.

Others Include haplotypes that had a frequency <10%.

## Discussion

In this case–control study of sporadic breast cancer in North Indian women we found that the variant genotypes rs2981582C/T, rs1219648A/G, rs2981578A/G and rs7895676T/C of *FGFR2* were all significantly associated with increased breast cancer risk. Recent identification of these intron 2 SNPs [17], [18] has drawn substantial attention towards *FGFR2* as a candidate gene for breast cancer. At present, much effort is focussed into targeting additional genetic alterations that drive breast cancer and *FGFR2* which has been implicated in different types of human malignancies, including breast cancer [33]–[36], is a likely candidate.

Since a previous report from South India [60] did not succeed in replicating the association of the studied *FGFR2* variant with breast cancer, as was observed in Europeans and other Asian populations [17], [46], [47], it was relevant to revisit the region along with other SNPs from the same LD block. The purpose of our study was to unravel any heterogeneity in association between population groups. Such differences reflect the variations among distinct geographic areas and ethnicity, and accentuate the necessity of characterizing breast cancer susceptibility genes among ethnic groups.

Present study reports significant association of rs2981582 and rs1219648 with breast cancer, consistent with previous observations from two Asian studies by Liang et al. [46] and Kawase et al. [47]. T allele of rs2981582 has been linked with an increased activity of *FGFR2* and it has been shown that haplotype marked by this allele associates with a higher level of *FGFR2* transcription both in breast cancer cell lines and tumors [56]. We observed an association of risk allele at rs2981582 and rs1219648 loci with breast cancer in premenopausal women, similar to some previous studies revealing the association of these variants with breast cancer risk in younger women [43]–[46]. We also observed rs2981582 T allele and rs1219648 G allele association with ER-positive than ER-negative tumors and further association with PR-positive than PR-negative tumors for rs2981582. Such findings of association with reproductive hormones are supported by several earlier studies showing that *FGFR2* variants contribute to breast cancer and confer their effect primarily in ER-positive and PR-positive tumor subtypes [42], [44], [46], [60], [61]. Also, higher levels of FGFR2 expression have been reported in ER-positive than ER-negative cell lines and tumors [62]–[64]. It is well known that elevated level of endogenous sex hormones, particularly estrogens, may increase breast cancer risk [65] and further, in premenopausal women exposure of endogenous serum estrogen is much higher as compared to post-menopausal women [66]. For rs2981582, we also observed an association of T allele with lower grade tumors, in accordance with a previous study by Garcia-Closas et al. [61]; with an early age at menarche (≤12 years), an observation previously reported by Kawase et al. [47]; and with employed status. Early onset of menarche is considered a breast cancer risk factor [67], [68] as early onset of menarche leads to early exposure of endogenous sex hormones and can induce proliferation of breast cells [67]. A significant proportion of breast cancer in India has been attributed to greater urbanization and changing life styles. Higher education and increased income have been shown to be as risk factors of breast cancer [69], [70].

For rs1219648, we observed a strong association of G allele with more invasive tumors with higher chance of LN metastasis, consistent with a previous report from China [54], and with clinically advanced stage, suggesting its association with disease aggressiveness. Moreover, we observed a very strong association of the risk allele with the use of exogenous hormones (either as contraceptives/infertility treatment/hormone replacement therapy), this is in somewhat contradiction to a previous report by Rebbeck et al. [53], where never users of combined hormone replacement therapy (CHRT) with the risk allele were at higher risk. Further, association with positive breastfeeding status was also observed. Exogenous hormone exposure and breastfeeding have been described as important factors predictive of breast cancer risk [71].

Breast cancer tends to be diagnosed at an earlier age in developing countries than in European and American populations and a rapid rate of increase in incidence has been observed before menopause [72]. Moreover, it has also been reported that premenopausal women constitute about 50% of all the breast cancer patients in India [6]. Thus, results from our study demonstrating the association of rs2981582 and rs1219648 with premenopausal status suggest the importance of investigating these two SNPs in Indian context. Moreover, restriction of the risk conferred by *FGFR2* variants to ER-positive and PR-positive tumors suggests that these SNPs affect the reproductive hormone-related pathway in the development of breast cancer in North Indian women. But these observations need to be confirmed in larger sample size studies from our population.

Recently done analysis by Meyer et al. have shown that two *FGFR2* SNPs rs2981578 and rs7895676 within intron 2 region alter the DNA binding affinity of transcription factors octamer-binding transcription factor 1 (Oct-1)/runt-related transcription factor 2 (Runx2) and CCAAT/enhancer binding protein β (C/EBPβ) respectively, resulting in an increased *FGFR2* gene expression both in cell lines and in breast tissues in patients homozygous for the risk allele as compared to those homozygous for the wild type allele [56]. These 2 SNPs are located in the same LD block of interest identified by GWAS [17], [18]. Role of these as breast cancer susceptibility variants is not yet established. In our study we observed a significant association of G allele of rs2981578 with breast cancer risk which is in accordance with a previous African American study [49] and a Chinese study [52]. We also observed an association of G allele with LN-positive status and with advanced clinical stage suggesting that the risk allele might relate to a more aggressive form of breast cancer. Further, association with parous status was observed. For rs7895676, we observed significant association of C allele with breast cancer risk, consistent with an earlier study by Boyarskikh et al. [50]. We further observed association of C allele with PR-positive, LN-positive, less malignant grade I+II tumors and negative breastfeeding status. Both parity and breastfeeding have been described as important factors linked to breast cancer risk [71]. Moreover, nulliparity and negative breastfeeding status have been linked with increased risk for breast cancer in Indian population [73], [74].

Association with exogenous hormone exposure and higher stage for SNPs rs1219648 and rs2981578 respectively, achieved statistical significance even after Bonferroni correction (*P*<0.000694), while other clinical features lost statistical significance, suggesting the importance of these SNPs in sub-categorized breast cancers in our population. But these observations need to be confirmed in further studies with larger sample size, to rule out false positive results and to establish intron 2 *FGFR2* SNPs as breast cancer susceptibility loci.

On conducting combined risk analysis in our study population of North Indian women relative risk of developing breast cancer is found to be elevated by around 65% for women carrying 2–4 risk loci as compared to the remaining groups carrying 0–1 risk loci (aOR = 1.645, 95%CI = 1.152–2.347). Moreover a progressively augmented risk with increasing number of risk loci was also noted demonstrating that a combination of these variants cumulatively increases risk (Table S3). In haplotype analysis, the *FGFR2* rs2981578 G/rs2981582 T/rs1219648 G haplotype was associated with a significantly increased breast cancer risk compared with the rs2981578A/rs2981582 C/rs1219648 A haplotype. Our findings on combinatorial effect of these loci and haplotype analysis are to several extent similar to previous studies [44]–[46], [51], [75], though they included only 2 or 3 *FGFR2* variants we are reporting here. Although, the tendency to increase breast cancer risk was significant across all the four SNPs tested, but the LD pattern between the four *FGFR2* variants in our North Indian population was weak to moderate only, in contrast to Europeans, but resembling other Asian populations [17], [46], [47], [75], indicating a fairly independent risk effect of each locus in our population, but the results warrant screening in larger sample sets. Moreover, we also observed significant differences in the contribution of different number of risk loci as well as different combinations of SNPs both in combined risk analysis as well as haplotype analysis, which resulted in varied extent of involvement towards risk.

To the best of our knowledge, we are reporting for the first time, a case control study on these four intronic *FGFR2* variants taken together along with LD measurement, haplotype analysis and stratified analysis for possible correlation with patients' clinical parameters in susceptibility to breast cancer.

Location of these *FGFR2* variants in intronic region suggests the probable explanation for their association with the risk through differential expression. Aberrant expression of alternatively spliced isoforms of *FGFR2* has been shown to activate signal transduction leading to transformation in breast cancer cells [76]. Variable expression of *FGFR2* in relation to intron 2 SNPs has been supported by the analysis carried out by Meyer et al. [56] and Huijts et al. [77]. Further, *FGFR2* intron 2 shows a high degree of conservation in mammals, and number of conserved putative transcription-factor binding sites have been identified in it [17], [78], some of which lie in close proximity to the significant SNPs. However, the exact mechanism of how these SNPs affect *FGFR2* upregulation remains unclear.

Besides SNPs, other features of *FGFR2* could be targeted in search for newer and efficient biomarkers in the future. Several altered FGFR2 characteristics have been linked with breast tumorigenesis and have shown promising results in studies on breast cancer cell lines and tumors, like amplification and overexpression, mutations, alternative splicing and isoform switching [33]–[38], [62], [76], [79]–[83]. Though, none of them has reached the clinical phase as yet and there are many hurdles to be overcome, there is enough encouraging evidence suggesting that targeting FGFR2 along with other FGFRs in certain subtypes of breast cancer could be a valuable approach in the future [84]–[88].

In conclusion, our study revealed a significant association of *FGFR2* intron 2 SNPs with breast cancer risk, as well as their interaction with various clinical parameters revealing their contribution to breast cancer susceptibility among North Indian women. Although, findings of the present study by themselves are unlikely to have any immediate clinical implications, however, such studies may play a key role in elucidating the biological mechanism that underline breast tumor heterogeneity, which may ultimately lead to improved treatment and prevention. These findings suggest that genetic variants of *FGFR2* might be used as candidate potential biomarkers for breast cancer risk. Further epidemiological and experimental studies of larger data sets along with sub-categorization by clinical parameters and expression studies are warranted to explore and confirm the role of these variants in increasing breast cancer risk, particularly from India, that will help us better understand the genetic heterogeneity in complex diseases like breast cancer.

## Supporting Information

Figure S1 **Linkage disequilibrium analysis {a) D′ value and b) r^2^ value} of the four intronic** ***FGFR2*** **SNPs in the studied population of North India.**
(TIF)

Table S1 **Clinical, lifestyle and other demographic details of cases (N = 368) and controls (N = 484) in the present study.**
(DOC)

Table S2 **Details of the SNPs selected for present study.**
(DOC)

Table S3 **Distribution of risk loci for the studied** ***FGFR2*** **SNPs (rs7895676, rs2981578, rs2981582 and rs1219648) in cases and controls of North India.**
(DOC)

Table S4 **One Way ANOVA for relative risk assessment of different number of risk loci for the studied** ***FGFR2*** **SNP combinations.**
(DOC)

Table S5 **Frequencies of inferred haplotypes of various** ***FGFR2*** **SNPs combinations taken two at a time in breast cancer cases and controls.**
(DOC)
