# Supplementary figures and images for: A Study on Genetic Variants of Fibroblast Growth Factor Receptor 2 (FGFR2) and the Risk of Breast Cancer from North India
Source: PLoS One. 2014 Oct 21;9(10):e110426. doi: 10.1371/journal.pone.0110426 (PMC4204868; doi:10.1371/journal.pone.0110426)

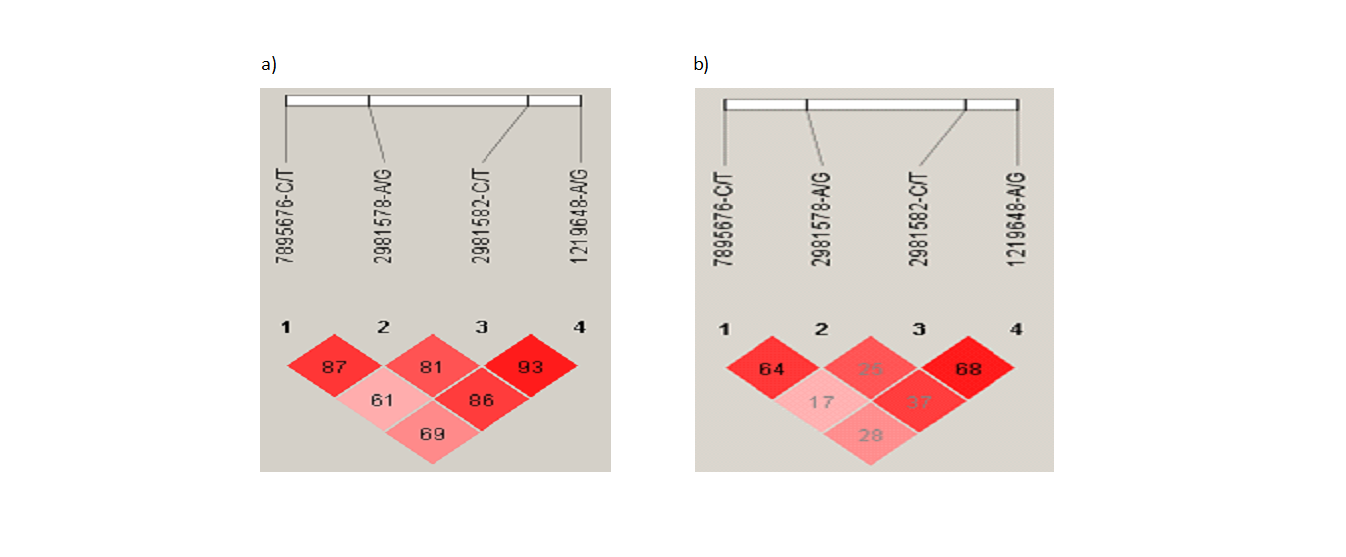

Supplement: Figure S1 — Linkage disequilibrium analysis {a) D′ value and b) r2 value} of the four intronic FGFR2 SNPs in the studied population of North India. (TIF) [file pone.0110426.s001.tif]
