# Supplementary material for: A Study on Genetic Variants of Fibroblast Growth Factor Receptor 2 (FGFR2) and the Risk of Breast Cancer from North India
Source: PLoS One. 2014 Oct 21;9(10):e110426. doi: 10.1371/journal.pone.0110426 (PMC4204868; doi:10.1371/journal.pone.0110426)
Supplement: Table S1 — Clinical, lifestyle and other demographic details of cases (N = 368) and controls (N = 484) in the present study. (DOC) [file pone.0110426.s002.doc]

**Supplementary Table S1:** Clinical, lifestyle and other demographic details of cases (N=368) and controls (N=484) in the present study.

| **Characteristic** | **Cases (%)** | **Controls (%)** |
| --- | --- | --- |
| Age (years) |  |  |
| ≤50 | 174 (47.28) | 227 (46.90) |
| >50 | 194 (52.72) | 257 (53.10) |
| Age at menarche (years) |  |  |
| ≤12 | 120 (32.61) | 145 (29.96) |
| >12 | 248 (67.39) | 339 (70.04) |
| Age at first live birth (years) |  |  |
| ≤29 | 290 (82.15) | 394 (87.17) |
| >29 | 63 (17.85) | 58 (12.83) |
| Parity |  |  |
| Nulliparous | 15 (4.08) | 32 (6.61) |
| Parous | 353 (95.92) | 452 (93.39) |
| Breastfeeding |  |  |
| Yes | 332 (90.22) | 431 (89.05) |
| No | 36 (9.78) | 53 (10.95) |
| Exogenous Hormone use |  |  |
| Yes | 53 (14.40) | 78 (16.12) |
| No | 303 (82.34) | 403 (83.26) |
| Unknown | 12 (3.26) | 3 (0.62) |
| Place of residence |  |  |
| Urban | 207 (56.25) | 275 (56.82) |
| Rural | 161 (43.75) | 209 (43.18) |
| Education level (years) |  |  |
| ≤12 | 169 (45.92) | 228 (47.11) |
| >12 | 192 (52.17) | 245 (50.62) |
| Unknown | 7 (1.90) | 11 (2.27) |
| Economic independence |  |  |
| Employed | 188 (51.09) | 266 (54.96) |
| Unemployed | 162 (44.02) | 206 (42.56) |
| Unknown | 18 (4.89) | 12 (2.48) |
| BMI (kg/m**^2^**) |  |  |
| <25 | 237 (64.40) | 353 (72.93) |
| ≥25 | 108 (29.35) | 114 (23.55) |
| Unknown | 23 (6.25) | 17 (3.51) |
| Menopausal status |  |  |
| Premenopausal | 155 (42.12) | 186 (38.43) |
| Postmenopausal | 213 (57.88) | 298 (61.57) |
| Age at menopause (years) |  |  |
| ≤ 49 | 98 (46.01) | 127 (42.62) |
| ≥ 50 | 115 (53.99) | 171 (57.38) |
| ER status |  |  |
| Positive | 172 (46.74) |  |
| Negative | 196 (53.26) |  |
| PR status |  |  |
| Positive | 179 (48.64) |  |
| Negative | 189 (51.36) |  |
| HER2 status |  |  |
| Positive | 174 (47.28) |  |
| Negative | 194 (52.72) |  |
| Tumor size (cm) |  |  |
| ≤2 | 99 (26.90) |  |
| >2 | 269 (73.10) |  |
| Lymph node |  |  |
| Positive | 225 (61.14) |  |
| Negative | 143 (38.86) |  |
| Clinical stage |  |  |
| I+II | 165 (44.84) |  |
| III+IV | 203 (55.16) |  |
| Histological grade |  |  |
| I+II | 253 (68.75) |  |
| III | 63 (17.12) |  |
| Unknown | 52 (14.13) |  |
