# Supplementary material for: A Study on Genetic Variants of Fibroblast Growth Factor Receptor 2 (FGFR2) and the Risk of Breast Cancer from North India
Source: PLoS One. 2014 Oct 21;9(10):e110426. doi: 10.1371/journal.pone.0110426 (PMC4204868; doi:10.1371/journal.pone.0110426)
Supplement: Table S2 — Details of the SNPs selected for present study. (DOC) [file pone.0110426.s003.doc]

**Supplementary Table S1**: Details of the SNPs selected for present study.

| **SNP** | **Chr:bp position** | **Primer positions, SNPs and restriction enzyme recognition sequences** | **Enzyme: recognition sequence** |
| --- | --- | --- | --- |
| rs2981582 | 10:123352317 | CGTGAGCCAAGCCTCTACTTGGTGCTGCACTGAAAT | AciI: |
|  |  | CTGTCATCAGTAGGGAATATTGGTAGCTGAGTTATT |  |
|  |  | TTTCGAGTGGTAATCCGAGAATAAAACGGCAGATCC | 5′...CCGC...3′ |
|  |  | CAGCACTCATCGCCACTTAATGAACCTGTTTGC/TGG | 3′...GGCG...5′ |
|  |  | AGAGTCCACCTGGTGCCTGCCTGGCTTTAGGAACCC |  |
|  |  | GCAGCAGTCCGAGTGGTGTCTGGGGTAAGCTGAGCT |  |
|  |  | GCTCTGGGAACACATCTCGTGCGTGGGGTGAATGAA |  |
|  |  | CAGCACACTTA |  |
| rs1219648 | 10:123346190 | ATGGTACCGGTTTCCCAAAACCAAAATTACTGAAAA | BspQI: |
|  |  | TCTAAAGCACGCCTATTTTACTTGACACACG/ACTCT |  |
|  |  | TCAAGGATGGCCATGGCTTGTCCCAATGATTCATAC | 5′...GCTCTTCN...3′ |
|  |  | TTGTGTATAAATATTTAAATGGTAAGAGCTTGAAGT | 3′...CGAGAAGNNNN...5′ |
|  |  | TATATAAATAGAAGTCAGCTACCACATACAAATCAC |  |
|  |  | A |  |
| rs2981578 | 10:123340311 | CCCAGAAAGCCTACATTCGTGGGAGCCGGCGCACA | AciI: |
|  |  | GCCCTTCTGAGATCTAAAGCTTCCCTCTGAATGCTGC |  |
|  |  | TTTGGAGGATTGTGAGAGGTAGTGACTCTTCAAAGT | 5′...CCGC...3′ |
|  |  | TTGTTTGTTTTCTTGAAGCTTTTACCTCTATGCAAAT | 3′...GGCG...5′ |
|  |  | ATGCG/AGTTTGGAGCAGGGAAGAAAGGTTAACTGT |  |
|  |  | GATGGCGCCGGCTCTTAACGTGGAATGTCCTGAATT |  |
|  |  | AATGTGGGTTTCAGTCCTCTGGCTCAGGATCCCCTG |  |
|  |  | AGGGAGAGTTTTTCTTTCCTCTGCAAAACACAGGAG |  |
|  |  | AAAAGTGATCCCTGTGGCTCCGACCTGCCTTCCTTG |  |
|  |  | GGTCCTG |  |
| rs7895676 | 10:123333997 | AGGTGCGGTGGCTCATGTCTGTAATACCAGCACTTT | DpnII: |
|  |  | GGGAGGCCCAGGCAGGTGGATCACTTGAGGTCAGG |  |
|  |  | AGTTCGAGACCGGCCTGGCCAACAAGACGAAACCC | 5′...GATC...3′ |
|  |  | CGTCTCTACTAAAAACACAAAAATTAGATGGGCATG | 3′...CTAG...5′ |
|  |  | GTGGTGTGTGCCTGTAGTCCCAGCTACACGGGAAGC |  |
|  |  | TGAGGCAGGAGAATTGCTTGAATCCGGGAGGCGGG |  |
|  |  | GGTTGCAGTGAGCCCAGATT/CGCACCACTGCACTCC |  |
|  |  | AGCCTGGGTGACAGAACTAAACTCCATCTCAAAAAT |  |
|  |  | AATAATAATAATAATAATTAAATTAAAAAATAAAG |  |
|  |  | GCAATGGAGTCCCGCCATTGAAGTCAG |  |

Primer sequences are highlighted in yellow colour, SNPs are marked with red colour, enzyme recognition sequences are underlined while cutting sites are indicated by bold arrows.
