# Supplementary material for: A Study on Genetic Variants of Fibroblast Growth Factor Receptor 2 (FGFR2) and the Risk of Breast Cancer from North India
Source: PLoS One. 2014 Oct 21;9(10):e110426. doi: 10.1371/journal.pone.0110426 (PMC4204868; doi:10.1371/journal.pone.0110426)
Supplement: Table S3 — Distribution of risk loci for the studied FGFR2 SNPs (rs7895676, rs2981578, rs2981582 and rs1219648) in cases and controls of North India. (DOC) [file pone.0110426.s004.doc]

| SNP Combi-nations | No. of risk loci | No. of cases (%) | No. of controls (%) | aOR (95% CI) | *P* value |
| --- | --- | --- | --- | --- | --- |
| **^d^**ABCD | 0 | 40 (10.87) | 88 (18.18) | 1.000 (Referent) |  |
|  | 1 | 16 (4.35) | 22 (4.55) | 1.600 (0.754-3.394) | 0.221 |
|  | 2 | 61 (16.58) | 75 (15.50) | 1.786 (1.076-2.964) | 0.025 |
|  | 3-4 | 251 (68.21) | 299 (61.78) | 1.855 (1.230-2.799) | 0.003 |
| ABC | 0 | 40 (10.87) | 88 (18.18) | 1.000 (Referent) |  |
|  | 1 | 29 (7.88) | 28 (5.79) | 2.302 (1.202-4.409) | 0.012 |
|  | 2 | 91 (24.73) | 133 (27.48) | 1.498 (0.942-2.381) | 0.088 |
|  | 3 | 208 (56.52) | 235 (48.55) | 1.959 (1.289-2.978) | 0.002 |
| BCD | 0 | 43 (11.68) | 96 (19.83) | 1.000 (Referent) |  |
|  | 1 | 62 (16.85) | 79 (16.32) | 1.738 (1.063-2.844) | 0.028 |
|  | 2 | 65 (17.66) | 67 (13.84) | 2.210 (1.330-3.671) | 0.002 |
|  | 3 | 198 (53.80) | 242 (50.00) | 1.824 (1.215-2.739) | 0.004 |
| ABD | 0 | 40 (10.87) | 87 (17.98) | 1.000 (Referent) |  |
|  | 1 | 22 (5.98) | 32 (6.61) | 1.485 (0.760-2.901) | 0.247 |
|  | 2 | 77 (20.92) | 85 (17.56) | 2.002 (1.223-3.278) | 0.006 |
|  | 3 | 229 (62.23) | 280 (57.85) | 1.782 (1.178-2.694) | 0.006 |
| ACD | 0 | 54 (14.67) | 101 (20.87) | 1.000 (Referent) |  |
|  | 1 | 58 (15.76) | 76 (15.70) | 1.430 (0.885-2.312) | 0.144 |
|  | 2 | 47 (12.77) | 76 (15.70) | 1.135 (0.684-1.883) | 0.624 |
|  | 3 | 209 (56.79) | 231 (47.73) | 1.704 (1.164-2.494) | 0.006 |

**Supplementary Table S3** Distribution of risk loci for the studied *FGFR2* SNPs (rs7895676, rs2981578, rs2981582 and rs1219648) in cases and controls of North India.

*OR* odds ratio, *CI* confidence interval

*P* value and corresponding age-adjusted OR (aOR) with 95% CIs for combined risk analysis by logistic regression test.

A= rs7895676, B = rs2981578, C = rs2981582 and D = rs1219648
