# Supplementary material for: A Study on Genetic Variants of Fibroblast Growth Factor Receptor 2 (FGFR2) and the Risk of Breast Cancer from North India
Source: PLoS One. 2014 Oct 21;9(10):e110426. doi: 10.1371/journal.pone.0110426 (PMC4204868; doi:10.1371/journal.pone.0110426)
Supplement: Table S4 — One Way ANOVA for relative risk assessment of different number of risk loci for the studied FGFR2 SNP combinations. (DOC) [file pone.0110426.s005.doc]

**Supplementary Table S4:** One Way ANOVA for relative risk assessment of different number of risk loci for the studied *FGFR2* SNP combinations.

1. Descriptive analysis

| SNP Combinations | No. of risk loci | N | Mean | Std. Deviation | Std. Error | 95% Confidence Interval for Mean | |
| --- | --- | --- | --- | --- | --- | --- | --- |
|  |  |  |  |  |  | Lower Bound | Upper Bound |
| ABCD | 0 | 128 | .31 | .465 | .041 | .23 | .39 |
|  | 1 | 38 | .42 | .500 | .081 | .26 | .59 |
|  | 2 | 136 | .45 | .499 | .043 | .36 | .53 |
|  | 3 | 119 | .41 | .494 | .045 | .32 | .50 |
|  | 4 | 431 | .47 | .500 | .024 | .42 | .52 |
|  | Total | 852 | .43 | .496 | .017 | .40 | .47 |
| ABC | 0 | 128 | .31 | .465 | .041 | .23 | .39 |
|  | 1 | 57 | .51 | .504 | .067 | .37 | .64 |
|  | 2 | 224 | .41 | .492 | .033 | .34 | .47 |
|  | 3 | 443 | .47 | .500 | .024 | .42 | .52 |
|  | Total | 852 | .43 | .496 | .017 | .40 | .47 |
| BCD | 0 | 139 | .31 | .464 | .039 | .23 | .39 |
|  | 1 | 141 | .44 | .498 | .042 | .36 | .52 |
|  | 2 | 132 | .49 | .502 | .044 | .41 | .58 |
|  | 3 | 440 | .45 | .498 | .024 | .40 | .50 |
|  | Total | 852 | .43 | .496 | .017 | .40 | .47 |
| ABD | 0 | 127 | .31 | .466 | .041 | .23 | .40 |
|  | 1 | 54 | .41 | .496 | .067 | .27 | .54 |
|  | 2 | 162 | .48 | .501 | .039 | .40 | .55 |
|  | 3 | 509 | .45 | .498 | .022 | .41 | .49 |
|  | Total | 852 | .43 | .496 | .017 | .40 | .47 |
| ACD | 0 | 155 | .35 | .478 | .038 | .27 | .42 |
|  | 1 | 134 | .43 | .497 | .043 | .35 | .52 |
|  | 2 | 123 | .38 | .488 | .044 | .30 | .47 |
|  | 3 | 440 | .48 | .500 | .024 | .43 | .52 |
|  | Total | 852 | .43 | .496 | .017 | .40 | .47 |

1. Multiple Comparisons output

| (I) ABCD | (J) ABCD | Mean Difference (I-J) | Std. Error | Sig. | 95% Confidence Interval | |
| --- | --- | --- | --- | --- | --- | --- |
|  |  |  |  |  | Lower Bound | Upper Bound |
| 0 | 1 | -.109 | .091 | .234 | -.29 | .07 |
|  | 2 | -.136^*^ | .061 | .026 | -.26 | -.02 |
|  | 3 | -.099 | .063 | .115 | -.22 | .02 |
|  | 4 | -.156^*^ | .050 | .002 | -.25 | -.06 |
| ABC |  |  |  |  |  |  |
| 0 | 1 | -.196^*^ | .079 | .013 | -.35 | -.04 |
|  | 2 | -.094 | .055 | .086 | -.20 | .01 |
|  | 3 | -.157^*^ | .049 | .002 | -.25 | -.06 |
| BCD |  |  |  |  |  |  |
| 0 | 1 | -.130^*^ | .059 | .027 | -.25 | -.01 |
|  | 2 | -.183^*^ | .060 | .002 | -.30 | -.07 |
|  | 3 | -.141^*^ | .048 | .003 | -.23 | -.05 |
| ABD |  |  |  |  |  |  |
| 0 | 1 | -.092 | .080 | .250 | -.25 | .07 |
|  | 2 | -.160^*^ | .059 | .006 | -.28 | -.05 |
|  | 3 | -.135^*^ | .049 | .006 | -.23 | -.04 |
| ACD |  |  |  |  |  |  |
| 0 | 1 | -.084 | .058 | .148 | -.20 | .03 |
|  | 2 | -.034 | .060 | .572 | -.15 | .08 |
|  | 3 | -.127^*^ | .046 | .006 | -.22 | -.04 |

*The mean difference is significant at the 0.05 level

A=rs7895676, B=rs2981578, C=rs2981582 and D=rs1219648
