# Supplementary material for: A Study on Genetic Variants of Fibroblast Growth Factor Receptor 2 (FGFR2) and the Risk of Breast Cancer from North India
Source: PLoS One. 2014 Oct 21;9(10):e110426. doi: 10.1371/journal.pone.0110426 (PMC4204868; doi:10.1371/journal.pone.0110426)
Supplement: Table S5 — Frequencies of inferred haplotypes of various FGFR2 SNPs combinations taken two at a time in breast cancer cases and controls. (DOC) [file pone.0110426.s006.doc]

**Supplementary Table S5:** Frequencies of inferred haplotypes of various *FGFR2* SNPs combinations taken two at a time in breast cancer cases and controls.

| SNP combinations | Haplotype | Cases (N=368) | Controls (N=484) | OR(95% CI) | *P* value |
| --- | --- | --- | --- | --- | --- |
| AB | TA | 0.373 | 0.425 | 1.000 (referent) |  |
|  | CG | 0.539 | 0.474 | 1.297 (1.058-1.590) | 0.013 |
|  | Others | 0.088 | 0.101 | 0.995 (0.702-1.410) | 1.000 |
| AC | TC | 0.403 | 0.436 | 1.000 (referent) |  |
|  | CT | 0.348 | 0.259 | 1.449 (1.153-1.822) | 0.002 |
|  | Others | 0.249 | 0.305 | 0.881 (0.696-1.117) | 0.306 |
| AD | TA | 0.387 | 0.439 | 1.000 (referent) |  |
|  | CG | 0.391 | 0.321 | 1.381 (1.109-1.720) | 0.004 |
|  | Others | 0.222 | 0.240 | 1.048 (0.816-1.346) | 0.749 |
| BC | AC | 0.366 | 0.425 | 1.000 (referent) |  |
|  | GT | 0.348 | 0.294 | 1.372 (1.092-1.724) | 0.007 |
|  | Others | 0.286 | 0.281 | 1.185 (0.936-1.502) | 0.165 |
| BD | AA | 0.373 | 0.429 | 1.000 (referent) |  |
|  | GG | 0.415 | 0.357 | 1.330 (1.071-1.652) | 0.011 |
|  | Others | 0.212 | 0.214 | 1.137 (0.879-1.472) | 0.356 |
| CD | CA | 0.542 | 0.608 | 1.000 (referent) |  |
|  | TG | 0.363 | 0.309 | 1.318 (1.070-1.623) | 0.011 |
|  | Others | 0.095 | 0.083 | 1.292 (0.915-1.824) | 0.155 |

*OR* odds ratio*, CI* confidence interval

*P* value and corresponding OR with 95% CI for Fisher’s exact test

A = rs7895676, B = rs2981578, C = rs2981582 and D = rs1219648
